# Therapeutic Suggestions During General Anesthesia Reduce Postoperative Nausea and Vomiting in High-Risk Patients – A *Post hoc* Analysis of a Randomized Controlled Trial

**DOI:** 10.3389/fpsyg.2022.898326

**Published:** 2022-07-15

**Authors:** Hartmuth Nowak, Alexander Wolf, Tim Rahmel, Guenther Oprea, Lisa Grause, Manuela Moeller, Katharina Gyarmati, Corinna Mittler, Alexandra Zagler, Katrin Lutz, Johannes Loeser, Thomas Saller, Michael Tryba, Michael Adamzik, Ernil Hansen, Nina Zech

**Affiliations:** ^1^Department of Anesthesiology, Intensive Care Medicine and Pain Therapy, University Hospital Knappschaftskrankenhaus, Ruhr University Bochum, Bochum, Germany; ^2^Kassel School of Medicine, Kassel, Germany; ^3^Department of Anesthesiology and Intensive Care Medicine, University Hospital, University of Cologne, Cologne, Germany; ^4^Department of Anesthesiology, University Hospital, Ludwig Maximilian University Munich, Munich, Germany; ^5^Department of Anesthesiology, University Hospital Regensburg, University of Regensburg, Regensburg, Germany; ^6^Clinic for Anesthesiology, Intensive Care Medicine, Emergency Medicine and Pain Therapy, Kassel Hospital, Kassel, Germany

**Keywords:** general anesthesia, hypnotherapy, patient communication, postoperative nausea and vomiting, therapeutic suggestions

## Abstract

Postoperative nausea and vomiting (PONV) are one of the most adverse events after general anesthesia, a distressing experience, and pose a risk to the patient. Despite advances in drug prophylaxis and PONV treatment, the incidence remains high and additional non-pharmacological treatments are needed. In this *post hoc* analysis of a recently published double-blind multicenter randomized controlled trial on the efficacy of intraoperative therapeutic suggestions on postoperative opioid dosage, we analyzed the effects of intraoperative therapeutic suggestions on PONV. We focus on patients with a high risk of PONV (Apfel risk score of 3–4) and distinguished early (first two postoperative hours) and delayed PONV (2–24 h). A total of 385 patients with a moderate or high risk for PONV were included. The incidence of early and delayed PONV was reduced (22.7–18.3 and 29.9–24.1%, respectively), without statistical significance, whereas in high-risk patients (*n* = 180) their incidence was nearly halved, 17.2 vs. 31.2% (*p* = 0.030) and 20.7 vs. 34.4% (*p* = 0.040), corresponding to a number needed to treat of 7 to avoid PONV. In addition, there was a significant reduction in PONV severity. In a multivariate logistic regression model, assignment to the control group (OR 2.2; 95% CI: 1.1–4.8) was identified as an independent predictor of the occurrence of early PONV. Our results indicate that intraoperative therapeutic suggestions can significantly reduce the incidence of PONV in high-risk patients. This encourages the expansion of therapeutic suggestions under general anesthesia, which are inexpensive and virtually free of side effects.

**Clinical Trial Registration:** German Clinical Trials Register, https://drks.de, registration number: DRKS00013800.

## Introduction

Since postoperative nausea and vomiting (PONV) are major adverse events after surgery under general anesthesia (Cohen et al., 1994; Apfel et al., 1999), effective interventions, which are able to reduce the incidence of PONV, have always been the subject of anesthesiologic research (Gan et al., 2020). In addition to the fact that PONV is a very distressing experience (Myles et al., 2000), it can have a direct impact on the patient’s outcome. The appearance of PONV poses a risk of severe complications such as suture dehiscence, aspiration, pneumonia, dehydration, hydroelectrolytic changes, esophageal rupture, and increased intracranial pressure (Bremner and Kumar, 1993; Schumann and Polaner, 1999; Samuels, 2013).

Early PONV within the first two postoperative hours with a relationship to volatile anesthetics can be distinguished from delayed PONV (Apfel et al., 2002). Despite advances in drug prophylaxis and treatment of PONV, the incidence remains high and is reported to be up to 30% in all postoperative patients and up to 80% in high-risk patients, which can be predicted by the presence of 3 or 4 factors of the Apfel simplified risk score, which include: female sex, non-smoking status, history of PONV or motion sickness, and/or use of postoperative opioids (Apfel et al., 1999). Therefore, in addition to drug treatment, non-pharmacological measures must also be considered to effectively reduce the incidence of PONV. One possible approach used in the past is the application of perioperative therapeutic suggestions, i.e., given pre- or postoperatively in hypnosis (Holler et al., 2021), or under general anesthesia intraoperatively. Suggestions are defined as verbal or non-verbal messages that the receiver involuntarily accepts and follows (Varga, 2013) and might therefore affect behavior, emotions, and autonomous body functions. Their effects can not only be subjectively recorded, but objectively measured and quantified (Zech et al., 2020, 2022). It is observed that even under general anesthesia, the central auditory pathway remains intact (Madler et al., 1991), and the perception of sounds and words is not interrupted (Hudetz, 2008; Sanders et al., 2012). However, several randomized controlled trials conducted on the effects of verbal suggestions given during general anesthesia in the past could only show very heterogeneous results (Rosendahl et al., 2016). These trials were small, heterogeneous in design, and conducted mainly in the 1990s and therefore did not reflect the current management of general anesthesia and PONV prophylaxis.

A recently published double-blind multicenter randomized controlled trial on the efficacy of intraoperative therapeutic suggestions showed a positive effect on postoperative opioid dosage and pain within the first 24 h after surgery, while for the incidence of PONV no differences were observed (Nowak et al., 2020). This study included patients at high and moderate risk for PONV. However, especially high-risk patients need a multimodal therapy approach to prevent PONV (Gan et al., 2020). Therefore, the question of whether intraoperative therapeutic suggestions influence PONV in these patients is of great interest and may have an impact on PONV management since therapeutic suggestions promise to be side-effect-free. Therefore, we conducted this *post hoc* analysis on the effect of intraoperative therapeutic suggestions on PONV after general anesthesia in patients with a high risk of PONV.

## Materials and Methods

### Patients and Study Design

Parts of this study were recently published and reported on the effects of therapeutic suggestions during general anesthesia on postoperative pain and opioid use (Nowak et al., 2020). This study was registered in the German Clinical Trials Register (registration number DRKS00013800, registration date 26th January 2018). In a double-blind randomized, placebo-controlled trial in 5 tertiary care hospitals in Germany, patients were included between the ages of 18 and 70 who underwent elective surgery requiring general anesthesia with a planned duration of 1–3 h and a risk of PONV, defined by an Apfel risk score (Apfel et al., 1999) of two or more points. Exclusion criteria were an American Society of Anesthesiologist (ASA) score of ≥4 (Owens et al., 1978), requirement for postoperative mechanical ventilation, or the use of regional anesthesia. Eligible patients were included after written informed consent.

### Ethics

The study was approved by the Ethics Committee of the Ruhr-University Bochum Medical Faculty, Bochum, Germany (Chairman Prof. Dr. M. Zenz, approval No. 17-5957-BR) on 15th May 2017.

### Study Procedures

**FIGURE 1:**
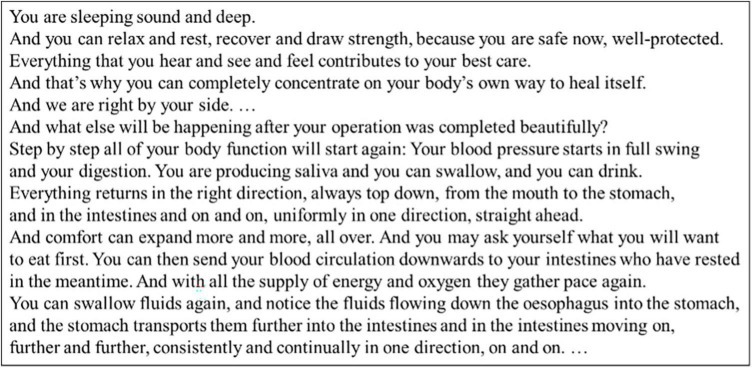
Example text of the therapeutic suggestions used in this study.

Patients were randomly assigned in a 1:1 ratio to intervention or control group. After induction of general anesthesia, patients in the intervention group listened to an Audio File containing background music and therapeutic suggestions, based on hypnotherapeutic principles, which included direct and indirect positive messages (see Figure 1). The tape was continuously played during surgery over earphones. At the end of surgery, a different file was presented to prepare the patients for emergence from anesthesia. Patients in the control group listened to a blank Audio File. For details see Nowak et al. (2020).

General anesthesia was performed as balanced anesthesia with volatile anesthetics (sevoflurane, isoflurane, or desflurane) at a minimum alveolar concentration of 1.0 ± 0.2 and repeated opioid administration. The depth of anesthesia was controlled by electroencephalography-based monitoring (Bispectral Index, Medtronic, Meerbusch, Germany, or Narcotrend, Narcotrend Group, Hannover, Germany), with a target index of 40–60. Both, a strict range of MAC above 0.8 and anesthesia depth monitoring, guarantee exclusion of inadequate anesthesia (Merikle and Daneman, 1996; Messina et al., 2016). Postoperative pain therapy was nurse or patient-controlled, according to local protocols. Before surgery, patients’ susceptibility to verbal suggestions was tested using a modified Harvard Group 5-item Hypnotic Susceptibility (HGSHS-5:G) (Riegel et al., 2021), and the level of anxiety was tested using the State Trait Anxiety Inventory (STAI-S) (Marteau and Bekker, 1992).

### Postoperative Nausea and Vomiting Management and Outcome Measures

The risk of PONV was assessed by the preoperative Apfel risk score (Apfel et al., 1999). Only patients with medium or high risk of PONV (2–4 points) were eligible for study inclusion. Pharmacological PONV prophylaxis was administered before general anesthesia induction or intraoperatively according to local standards. These included, among others, dexamethasone, ondansetron, droperidol, metoclopramide, or dimenhydrinate. After surgery, the incidence of PONV was evaluated in the recovery room (first 2 h) and 24 h after extubation (normal ward). The severity of PONV was assessed using the simplified PONV impact scale (0–6), described by Myles and Wengritzky (2012). Treatment of PONV was performed again with dexamethasone, ondansetron, droperidol, metoclopramide, dimenhydrinate, or a combination. Antiemetic milligram equivalents (AMEs) were calculated for the comparability of various antiemetics (AME = ondansetron × 4 + dexamethasone × 4 + droperidol × 1.25 + metoclopramide × 20 + dimenhydrinate × 50) (Apfel et al., 2004a).

### Statistics

Sample size calculation was carried out on the primary outcome (postoperative opioid use), which was based on a recent meta-analysis (Rosendahl et al., 2016). Based on a 1:1 randomization ratio with an assumed effect size of 0.3, we calculated a total of 368 patients to obtain 80% power to detect a difference in postoperative opioid dosage at a two-sided α level of 0.05. Baseline characteristics and outcomes were analyzed as follows: continuous variables are presented as mean ± standard deviation (SD) for normally distributed variables and median and interquartile range (IQR; 25th and 75th percentiles) for non-normally distributed variables. Categorical variables are expressed as frequency and percentage. Comparison of continuous variables between groups was performed using a parametric Student’s *t*-test or a non-parametric Mann–Whitney *U*, respectively. Categorical variables were compared using Pearson’s Chi-square test and by calculation of the number needed to treat (NNT). In addition, the resulting risk differences including the 95% confidence intervals were calculated. In contrast to the previously published data of this study we performed, for better assessment of non-normally distributed variables in the outcome analysis, a bootstrapping method with resampling and calculated the means, SD, and 95% confidence intervals. For further analysis, we *post hoc* formed a subgroup of patients at high risk of PONV, defined by a preoperative Apfel score of 3–4. For the assessment of the joint effect of therapeutic suggestions and potential confounding factors in this subgroup, a logistic regression analysis was performed with single and multiple predictors. These included: assignment to the control group, preoperative Apfel score, dose of intraoperative antiemetics and opioids, type of surgery, and duration of surgery. Finally, a multivariable restricted model was built by using stepwise backward elimination. Furthermore, since the severity of PONV and the application of antiemetics are not independent variables, their correlation was analyzed represented by the Spearman coefficient. Statistical analysis was performed with The R Project for Statistical Computing 4.0.4 (The R Foundation for Statistical Computing, Vienna, Austria). Graphical representations of the results were created with GraphPad Prism 8.1 (GraphPad Software, San Diego, CA, United States). A two-sided *p*-value of less than 0.05 was considered statistically significant.

## Results

**FIGURE 2:**
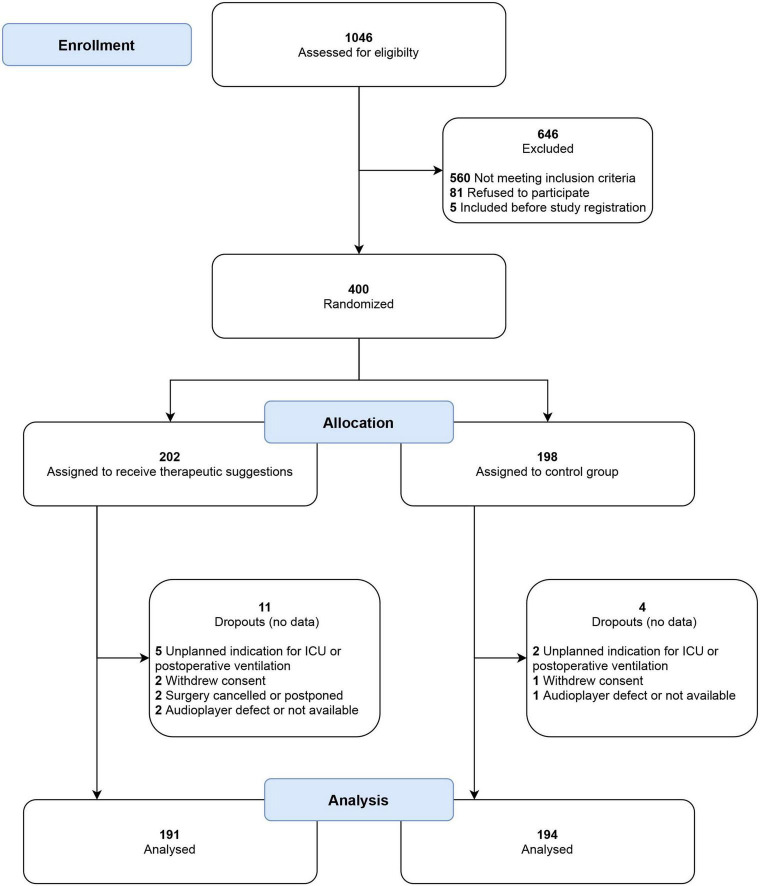
CONSORT flow chart of patient recruitment. No postoperative data were collected for dropouts, and they were excluded from analysis before unblinding of the study. ICU, intensive care unit.

In total, 400 patients were recruited and randomized from January to December 2018 and 385 of them were analyzed in the per-protocol analysis (191 in intervention and 194 in control group), see Figure 2. The subgroup of patients with a high risk of PONV is formed by 180 patients, 87 patients in the intervention and 93 patients in the control group. Table 1 presents the baseline characteristics. Almost all parameters for the entire cohort, as well as for the high-risk subcohort, were evenly distributed between both groups. Only the distribution of the types of surgery in high-risk patients was uneven, with a higher proportion of intraabdominal surgeries in the control group and a resulting lower proportion of other types of surgery. None of the patients reported remembering to wear headphones or listening to music or verbal suggestions. No side effects were observed.

**TABLE 1:** Baseline characteristics for all patients and subgroup of patients at high risk for postoperative nausea and vomiting, defined by pre-operative Apfel-score of 3 or 4.

	All patients (*n* = 385)			Patients at high risk for PONV^1^ (*n* = 180)		
		
	Intervention group (*n* = 191)	Control group (*n* = 194)	*p*	Intervention group (*n* = 87)	Control group (*n* = 93)	*p*
Age (years), median (IQR)	52 (43–62)	54 (46–62)	0.241	52 (43–62)	53 (46–61)	0.708
Female sex, *n* (%)	115 (60.2)	110 (56.7)	0.484	73 (83.9)	71 (76.3)	0.205
**Pre-operative score results, median (IQR)**						
Apfel score^2^	2 (2–3)	2 (2–3)	0.688	3 (3–3)	3 (3–3)	0.683
HGSHS-5^3^	1 (0–3)	1 (0–3)	0.798	2 (0–4)	1 (0–3)	0.483
STAI-S^4^	41 (33–51)	40 (33–50)	0.478	43 (33–52)	44 (34–53)	0.937
NRS^5^	0 (0–1)	0 (0–2)	0.308	0 (0–0)	0 (0–0)	0.396
**Type of surgery, *n* (%)**						
Intra-abdominal^6^	61 (31.9)	77 (39.7)	0.489	20 (23.0)	32 (34.4)	0.040
Thyroid gland	36 (18.8)	30 (15.5)		24 (27.6)	16 (17.2)	
Gynecological^7^	24 (12.6)	15 (17.7)		21 (24.1)	11 (11.8)	
Urogenital^8^	21 (11.0)	26 (13.4)		5 (5.7)	12 (12.9)	
Other^9^	49 (25.7)	46 (13.7)		17 (19.6)	22 (23.7)	
Duration of surgery (min), median (IQR)	95 (69–140)	106 (74–141)	0.144	91 (68–128)	113 (74–135)	0.113
**Intra-operative drug use**						
Fentanyl (mg)^10^, median (IQR)	0.5 (0.4–0.5)	0.5 (0.5–0.6)	0.148	0.5 (0.4–0.5)	0.5 (0.4–0.6)	0.210
Sufentanil (μg)^11^, median (IQR)	50 (40–64)	50 (40–70)	0.232	50 (39–60)	50 (40–62)	0.494
PONV prophylaxis^12^, *n* (%)	94 (49.2)	99 (51.0)	0.722	55 (63.2)	61 (65.6)	0.740

*^1^High risk for postoperative nausea and vomiting (PONV) is defined by a pre-operative Apfel score of 3 or 4 points. ^2^Apfel score: Apfel score of risk for postoperative nausea and vomiting (0–4). ^3^HGSHS-5: 5-item version of Harvard Group Scale for Hypnotic Susceptibility (0–5). ^4^STAI-S: State Trait Anxiety Inventory Scale (20–80). ^5^NRS: numeric rating scale of pain (0–10). ^6^Inter alia gastric surgery, colorectal surgery, hepatic surgery, cholecystectomy.^7^Inter alia hysterectomy, ovariectomy, pelvic floor repair.^8^Inter alia prostatectomy, bladder surgery. ^9^Inter alia herniated intervertebral disc, lumbar spinal stenosis, adrenalectomy, plastic/reconstructive surgery. ^10^n = 85/93 (intervention/control group) for all patients and n = 47/50 (intervention/control group) for patients at risk for PONV. ^11^n = 106/101 (intervention/control group) for all patients and n = 40/43 (intervention/control group) for patients at risk for PONV. ^12^Intraoperative, preventive medication against postoperative nausea and vomiting (PONV) with ondansetron, dexamethasone, droperidol, metoclopramide, dimenhydrinate, or a combination according to local protocols of each study site. The following missing data were excluded from the analysis: 109/67 cases missing (all patients/patients at risk for PONV) for preoperative HGSHS-5 score.*

### Incidence and Severity of Postoperative Nausea and Vomiting

**FIGURE 3:**
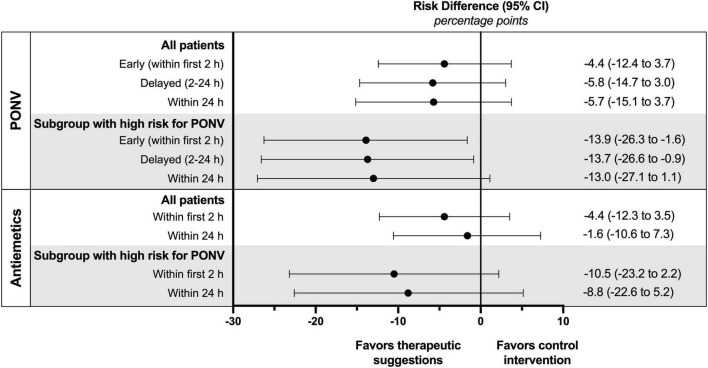
Absolute risk differences of PONV incidence and postoperative use of antiemetics for all patients and subgroup of patients with a high risk for PONV (defined by an Apfel score of 3 or 4).

Outcomes are presented in Table 2. In the cohort of all patients no significant differences were observed in the incidence or severity of PONV. If the focus is set on patients at high risk for PONV, intraoperative therapeutic suggestions showed a marked effect. The incidence of both early and delayed PONV was significantly reduced by 45 or 40%, respectively (Figure 3). This corresponds to the number of patients needed to treat (NNT) of approximately 7 to avoid one case of early or delayed PONV. For high-risk patients, the PONV impact score was reduced by the intervention by approximately 50% within the first 2 or 24 h.

**TABLE 2:** Outcome variables for all patients and subgroup of patients at high risk for postoperative nausea and vomiting, defined by a pre-operative Apfel-score of 3 or 4.

	All patients (*n* = 385)				Patients at high risk for PONV^1^ (*n* = 180)			
		
	Intervention group (*n* = 191)	Control group (*n* = 194)	*p*	NNT^2^	Intervention group (*n* = 87)	Control group (*n* = 93)	*p*	NNT
**PONV^3^, *n* (%)**								
Early (within first 2 h)	35 (18.3)	44 (22.7)	0.290	23.0	15 (17.2)	29 (31.2)	0.030	7.1
Delayed (2–24 h)	46 (24.1)	58 (29.9)	0.199	17.2	18 (20.7)	32 (34.4)	0.040	7.3
Within 24 h	59 (30.9)	71 (36.6)	0.236	17.5	28 (32.2)	42 (45.2)	0.074	7.7
**PONV impact scale score^4^, mean ± SD**								
Within first 2 h	0.20 ± 0.52	0.26 ± 0.66	0.289	–	0.16 ± 0.43	0.33 ± 0.78	0.039	–
Within 24 h	0.42 ± 0.88	0.55 ± 1.09	0.173	–	0.36 ± 0.88	0.74 ± 1.33	0.017	–
**Postoperative use of antiemetics, *n* (%)**								
Within first 2 h	33 (17.3)	42 (21.6)	0.279	22.9	18 (20.7)	29 (31.2)	0.109	9.5
Within 24 h	51 (26.7)	55 (28.4)	0.717	60.6	27 (31.0)	37 (39.8)	0.220	11.4
**Postoperative AME^5^, mean ± SD**								
Within first 2 h	0.25 ± 0.66	0.30 ± 0.66	0.487	–	0.30 ± 0.66	0.49 ± 0.83	0.081	–
Within 24 h	0.42 ± 0.84	0.47 ± 0.90	0.524	–	0.47 ± 0.85	0.75 ± 1.11	0.073	–
Within first 2 h, in patients with use of antiemetics^6^	1.45 ± 0.89	1.37 ± 0.73	0.695	–	1.48 ± 0.61	1.56 ± 0.74	0.703	–
Within 24 h, in patients with use of antiemetics^7^	1.54 ± 0.93	1.66 ± 0.95	0.550	–	1.53 ± 0.86	1.87 ± 0.99	0.142	–

*Hours refer to timepoint after admission to recovery room. Means, standard deviations, and 95% CIs of non-normally distributed data were calculated by bootstrapping procedure. ^1^High risk for postoperative nausea and vomiting (PONV) is defined by a pre-operative Apfel score of 3 or 4 points. ^2^NNT: number needed to treat. ^3^PONV: postoperative nausea and vomiting (defined as patient reporting nausea or vomiting within the specified time interval). ^4^PONV impact scale score (0–6) by Myles and Wengritzky (2012). ^5^AME: antiemetic milligram equivalents = ondansetron × 4 + dexamethasone × 4 + droperidol × 1.25 + metoclopramide × 20 + dimenhydrinate × 50. ^6^Amount of antiemetics within first 2 h. ^7^Amount of antiemetics during first 24 h.*

### Use and Dose of Antiemetics

In patients with a high risk of PONV therapeutic suggestions resulted in an absolute risk reduction for the use of postoperative antiemetics of 11% within the first 2 h and 9% within 24 h, although not reaching statistical significance (Table 2 and Figure 3). The corresponding NNT was about 10. There was also a trend to a lower dose of postoperative antiemetics by one-third in the intervention group.

### Correlation of Postoperative Nausea and Vomiting Score and Dose of Antiemetic Milligram Equivalent

In the control group, a small to intermediate but significant correlation between the PONV score and the total antiemetics dose (intraoperative and postoperative) was observed for both time periods. With the intervention, this correlation decreased (Table 3). Furthermore, in patients who developed PONV, the correlation between PONV and the use of antiemetic lost statistical significance.

**TABLE 3:** Correlation between PONV severity and dose of antiemetics.

	Intervention group			Control group		
		
	Rho^1^	95% CI	*p*	Rho	95% CI	*p*
**Patients at risk for PONV^2^ (Apfel score 3–4)**						
Intraoperative + first 2 h	0.295	0.090–0.476	0.006	0.469	0.294–0.614	<0.001
Intraoperative + first 24 h	0.317	0.114–0.494	0.003	0.415	0.231–0.570	<0.001
**Patients with PONV**						
Intraoperative + first 2 h	0.325	−0.055 to 0.623	0.092	0.530	0.269–0.718	<0.001
Intraoperative + first 24 h	0.050	−0.329 to 0.415	0.801	0.401	0.111–0.629	0.008

*PONV severity according to Wengritzky score, antiemetics standardized to antiemetic milligram equivalents (AMEs). ^1^Rho: Spearman correlation coefficient. ^2^PONV: postoperative nausea and vomiting.*

### Confounding Factors

Several factors associated with PONV were tested using logistic regression analysis (Table 4). In the univariate testing, the assignment to the control group had an impact on both early and delayed PONV. Further significant confounders were the preoperative Apfel risk score and intraabdominal surgery for early PONV, and the dose of postoperative opioids for delayed PONV. In the restricted multivariable model, assignment to the control group, preoperative Apfel score, and intraabdominal surgery were confirmed as independent predictors for the development of early PONV. For delayed PONV, only the PONV risk score and the postoperative dose of opioids remained predictors of statistical significance.

**TABLE 4:** Logistic regression model for single and multiple predictors of postoperative nausea and vomiting in patients with high risk (Apfel score 3–4) (*n* = 180).

	Univariable models			Multivariable models					
		
				Unrestricted			Restricted		
					
	OR	95% CI	*p*	OR	95% CI	*p*	OR	95% CI	*p*
**Early PONV^1^**									
No therapeutic suggestions	2.18	1.08–4.51	0.032	2.23	1.05–4.92	0.041	2.26	1.09–4.88	0.032
PONV risk score^2^	2.68	1.23–5.80	0.012	3.31	1.40–7.92	0.007	3.08	1.36–7.02	0.007
PONV prophylaxis^3^	1.22	0.84–1.80	0.297	1.13	0.70–1.81	0.618	–	–	–
Intraoperative fentanyl	1.15	0.35–3.53	0.812	1.78	0.16–19.25	0.632	–	–	–
Intraoperative sufentanil	1.00	0.99–1.02	0.542	1.00	0.98–1.03	0.771	–	–	–
Type of surgery									
Intra-abdominal	2.08	1.01–4.24	0.045	3.00	1.00–10.05	0.059	2.66	1.17–6.20	0.021
Thyroid gland	1.23	0.54–2.68	0.611	2.06	0.60–7.60	0.259	2.05	0.80–5.22	0.131
Gynecological	0.52	0.17–1.34	0.207	0.91	0.19–4.05	0.897	–	–	–
Urogenital	0.95	0.26–2.85	0.927	1.81	0.36–8.45	0.454	–	–	–
Duration of surgery	1.00	1.00–1.01	0.337	1.00	0.99–1.01	0.874	–	–	–
Opioid dosage^4^ within first 2 h	1.03	0.94–1.12	0.493	0.97	0.88–1.07	0.600	–	–	–
**Delayed PONV**									
No therapeutic suggestions	2.01	1.04–4.00	0.042	1.78	0.84–3.87	0.138	1.74	0.83–3.73	0.150
PONV risk score	2.10	0.97–4.48	0.055	2.59	1.08–6.27	0.032	2.43	1.04–5.68	0.040
PONV prophylaxis	1.31	0.91–1.89	0.151	1.02	0.61–1.67	0.928	–	–	–
Intraoperative fentanyl	0.44	0.13–1.37	0.170	2.10	0.15–24.09	0.560	–	–	–
Intraoperative sufentanil	1.01	1.00–1.02	0.031	1.01	0.99–1.04	0.372	–	–	–
Type of surgery									
Intra-abdominal	1.59	0.78–3.18	0.194	1.43	0.48–4.48	0.525	–	–	–
Thyroid gland	2.08	0.98–4.35	0.053	2.63	0.83–8.93	0.109	1.85	0.80–4.25	0.147
Gynecological	0.22	0.05–0.67	0.017	0.49	0.09–2.29	0.384	0.31	0.07–1.02	0.081
Urogenital	1.09	0.33–3.13	0.874	1.62	0.34–7.14	0.530	–	–	–
Duration of surgery	1.00	0.99–1.01	0.913	0.99	0.98–1.00	0.127	–	–	–
Opioid dosage within 24 h	1.10	1.05–1.16	<0.001	1.08	1.02–1.15	0.009	1.08	1.03–1.14	0.004

*Hours refer to timepoint after admission to recovery room. Restricted models were built by stepwise backward elimination. ^1^PONV: postoperative nausea and vomiting. ^2^PONV risk score: Apfel score of risk for postoperative nausea and vomiting (0–4). ^3^Antiemetic milligram equivalents = ondansetron × 4 + dexamethasone × 4 + droperidol × 1.25 + metoclopramide × 20 + dimenhydrinate × 50. ^4^Morphine milligram equivalents = piritramide × 0.7 + tilidine × 0.2 + oxycodone × 0.8.*

## Discussion

Postoperative nausea and vomiting is one of the most common adverse events after surgery under general anesthesia and has a profound impact on patient comfort and satisfaction (Myles et al., 2000). Patients are often more compromised by PONV than by postoperative pain (Simanski et al., 2001). Therefore, in addition to pharmacological options, effective non-pharmacological prophylaxis and treatments are urgently needed to reduce the incidence of PONV, especially in high-risk patients with a dramatically high incidence between 61 and 79% (Gan et al., 2020).

### Incidence, Severity, and Treatment of Postoperative Nausea and Vomiting

Our intervention was able to significantly reduce the incidence of both, “early” and “delayed” PONV in patients at high risk. Furthermore, PONV severity was halved by the intervention. However, the mean intensity of PONV was low, probably because more than half of these patients, although at risk, did not develop PONV. The low incidence and severity might be attributable to the wide use of pharmacological PONV prophylaxis in this study and to an accompanying placebo effect. The demand for antiemetics was reduced by approximately one-third. In general, whenever a dose is observed, the proportion of patients treated must also be considered. In our study, the number of patients with demand for antiemetics was also reduced by one-third. However, in these patients, the difference in the total antiemetic dose between the intervention and control groups diminished. Therefore, the main reason for the observed dose reduction was probably the decreased number of patients with demand for antiemetic treatment.

### Correlation Between Postoperative Nausea and Vomiting Severity and Antiemetic Dose

The use of antiemetics affects the severity of PONV, and the severity of PONV triggers the use and dose of antiemetics. Therefore, both factors must be considered. The intervention in our study affected and reduced the correlation of these two entities. A possible interpretation of these results is the induction of tolerance against PONV by intraoperative positive suggestions, where an identical dose of antiemetics results in lower manifestations of PONV in the intervention group, and a comparable severity of PONV leads to a lower requirement for antiemetic treatment. This development of tolerance by a change in the perception, the impact and the significance of nausea is one possible basis of the observed effects. Others are an antiemetic effect of the suggestions, including images of physiological functions (the idea of appetite or of a flow downward), or interference with the generation of nausea. In contrast to awake interventions the suggestions were given during surgery, i.e., at the time of surgery and anesthesia that might be responsible for the development of nausea.

### Predictors of Postoperative Nausea and Vomiting

The risk of PONV in adults is influenced by many different patient-specific and surgery-related factors, e.g., female sex, history of PONV, motion sickness, non-smoking status, young age, duration of surgery/anesthesia, and specific types of intraabdominal surgery (Apfel et al., 1999, 2004b,^2012^; Sinclair et al., 1999). Furthermore, anesthesia-related predictors of PONV include volatile anesthetics, nitrous oxide, and postoperative use of opioids, while these factors are dose and duration dependent (Apfel et al., 2002; Breitfeld et al., 2003; Habib et al., 2006; Myles et al., 2007; Peyton and Wu, 2014). As a result, current guidelines define different prophylactic measurements depending on the individual risk of PONV. These include, in addition to pharmacological antiemetic prophylaxis, the avoidance of volatile anesthetics and nitrous oxide, and instead the use of total intravenous anesthesia (TIVA) (Gan et al., 2020). Despite differentiated prophylactic therapy approaches, a large number of especially high-risk patients still suffer from PONV (Rusch et al., 2010), with a request for multimodal approaches that also include non-pharmacological interventions (Gan et al., 2020).

In the present study, assignment to the control group was a significant determinator of PONV in high-risk patients, which subsequently proved that intraoperative therapeutic suggestions are a promising intervention against PONV. Early PONV within the first 2 h after surgery was affected by the affiliation of the study group, the preoperative Apfel PONV risk score, and the type of surgery, namely intraabdominal operations. Especially the low impact of intraoperative opioid dose and duration of surgery for the incidence of early PONV is unexpected, as the respective dose of inhalational anesthetics was previously described as a determinator (Apfel et al., 2002). In contrast to the first 2 h after surgery, where patients were in a very controlled setting in the recovery room, delayed PONV was only affected by postoperative opioid dose. These findings may be attributable to the circumstances in the postoperative setting after discharge from the recovery room to the normal ward, where many other possible confounders occur that have not been recorded or evaluated. As both state anxiety and hypnotic susceptibility did not differ in the two groups, these parameters were not included in the multivariate analysis, and no conclusion on their impact can be drawn

### Comparison With Other Studies

Several different medical interventions have been tested and reported in the effort to prevent PONV (Gan et al., 2020). To avoid one case of PONV after isoflurane anesthesia, six patients would have to receive TIVA (Visser et al., 2001). Pharmacological antiemetic prophylaxis with ondansetron has a NNT of 6 for the prevention of vomiting and 7 for nausea, respectively (Tramer et al., 1997). In addition, non-pharmacological means including acupuncture were found effective (Lee et al., 2015). However, mainly antiemetics have found their way into the everyday clinical practice of PONV prophylaxis established in the meantime.

The effect of communication techniques on PONV has also been evaluated, for instance, by studies with perioperative hypnotherapy in awake patients (Kekecs et al., 2014) and under the rather special condition of general anesthesia (Rosendahl et al., 2016). In a meta-analysis of Rosendahl et al. (2016), only 3 out of 21 included trials showed a positive effect on the incidence of PONV. However, the overall effect identified therapeutic suggestions to significantly reduce PONV. Since these former studies were conducted mainly in the 1980s and 1990s without routine PONV prophylaxis, incidence, and severity of PONV were higher. However, our study in patients after PONV prophylaxis demonstrated an even higher effect with a NNT of 7.

In general, the comparison of the various pharmacological and non-pharmacological attempts of PONV prophylaxis shows that the effect of therapeutic suggestions in our study is of a comparable magnitude with much lower costs, effort, and side effects. It should be noted that in clinical practice often a combination of different treatments is necessary to achieve a sufficient antiemetic effect and that the result is additive (Weibel et al., 2021). In our study, almost all patients had received pharmacological PONV prophylaxis. However, the effect was measured against a control group with only antiemetic drugs. Thus, it can be assumed that the observed reduction in PONV is a direct consequence of the intervention tested. Therefore, therapeutic suggestions could provide an inexpensive and safe possibility for supplementation of PONV prophylaxis.

### Limitations

This study has several limitations. First of all, this is a *post hoc* analysis of an original study, therefore, our findings should be tested in a prospective, sufficiently powered study. Moreover, the role of other contributing factors than therapeutic suggestions remain unclear – for example, positive effects may also be expected from background music. Although regularly positive effects of music on pain and anxiety can be observed, mainly with treatment in awake patients (Cepeda et al., 2006; Hole et al., 2015; Kuhlmann et al., 2018), evidence for an antiemetic effect is missing (Stoicea et al., 2015). Furthermore, a beneficial effect can also be expected from shielding the ears from intraoperative noise and careless talk, including negative suggestions (Hansen and Zech, 2019). However, this was applicable to both groups in our study.

### On the Underlying Mechanism

The perception of words under general anesthesia was not unexpected, as evidence has been gathered that the auditory pathway is preserved during anesthesia (Madler et al., 1991; Hudetz, 2008). Moreover, the phenomenon of “intraoperative awareness” has been described regularly (Millar and Watkinson, 1983; Ghoneim and Block, 1992; Schwender et al., 1994). However, our results cannot be explained by a few patients reacting like in “intraoperative awareness” with an incidence of only 0.1–0.2% for explicit recall (Sanders et al., 2012) and a few percent for implicit memory (Fu et al., 2021; Linassi et al., 2021). Therefore, auditory impressions that a patient perceives under general anesthesia must be critically questioned, since conversations and noises in the operating room can have a negative influence on patients and should be avoided (Hansen and Zech, 2019).

With regard to the mechanisms responsible for the observed responses, we consider a reduced resistance to suggestions after loss of critical, rational thinking and an access to the subconscious to be responsible. This parallels the mechanism of hypnosis that is characterized by a depression of the dorsolateral frontal cortex (DLPFC) (Dienes and Hutton, 2013), and shows similar beneficial effects of suggestions in surgical patients (Kekecs et al., 2014). The difference between low incidences of “intraoperative awareness” and the high incidence of perception in this study may be explained by the content. While it is random in explicit memory (thoughtless conversations in the operating room) and neutral in experiments of implicit memory (test texts), it is characterized by meaning in the application of therapeutic communication before or during surgery. In experiments with intraoperative simulation of a ventilation incident, 8 out of 10 patients had an implicit memory or reaction (Levinson, 1965). While the reports on “intraoperative awareness” with its low incidences did not lead to a general change in the behavior in the operating rooms over all those years, hopefully the present demonstration of intraoperative perception will.

Intraoperative therapeutic suggestions were demonstrated to affect postoperative pain and request for analgesics (Nowak et al., 2020), as well as PONV and use of antiemetics as reported here. The high efficacy of the tested intervention compared to previous trials might be attributed to the specific text of the suggestions. Negative words and negations such as “no nausea” were avoided. Instead, “increased comfort,” appetite and pleasurable food intake after surgery were addressed. The suggestions presented intraoperatively dealt with items such as support, care, and self-healing power. From a text addressing such general topics of well-being, further effects can be expected and should be studied. Some interesting parameters that cannot be monitored and measured so fast and easily might be affected concurrently, such as wound healing, homeostasis, or immune surveillance, but also could be addressed more specifically.

We consider the addressing of themes of meaning essential for the observed effects, namely accompaniment, contact, comfort, confidence, information, control, instructions, respect, safety, and healing (Hansen and Zech, 2019). Constructing placebo effects as a mechanism of action is difficult since generation of expectations under general anesthesia has not been described yet. However, it has been suggested to better call the placebo effect a “meaning response” as well (Moerman and Jonas, 2002). Actually, response to meaning could be the common basis of hypnosis, therapeutic communication and placebo effects. The melody of the voice and the perception of a caring person close may play a role in addition.

## Conclusion

Our results encourage the use of therapeutic suggestions under general anesthesia, especially since it is an inexpensive intervention that is virtually free of side effects. They should not be limited to taped recordings favorable for standardized study conditions but stimulate personal talk to patients and wider application of positive and therapeutic communication also in awake patients. The demonstrated positive effects of therapeutic suggestions even under general anesthesia should stimulate further research and application in other patients during impaired wakefulness, such as during resuscitation or intensive care, “touching the unconscious in the unconscious.”

## Data Availability Statement

The raw data supporting the conclusions of this article will be made available by the authors, without undue reservation.

## Ethics Statement

The studies involving human participants were reviewed and approved by the Ethics Committee of the Ruhr-University Bochum Medical Faculty, Bochum, Germany. The patients/participants provided their written informed consent to participate in this study.

## Author Contributions

HN: writing of the manuscript, data analysis, and critical revision of the manuscript. AW and TR: data analysis and critical revision of the manuscript. GO: study conception and design, study center supervision, data collection, and critical revision of the manuscript. LG, MM, KG, CM, AZ, and KL: data collection, analysis and interpretation, and critical revision of the manuscript. JL, TS, and MT: study center supervision, data collection, analysis and interpretation, and critical revision of the manuscript. MA: study supervision and critical revision of the manuscript. EH and NZ: study conception and design, development and taping of the intervention text, study supervision, writing of the manuscript and data interpretation, and critical revision of the manuscript. All authors approved the final version to be published and agreed to be accountable for all aspects of the work.

## Conflict of Interest

The authors declare that the research was conducted in the absence of any commercial or financial relationships that could be construed as a potential conflict of interest.

## Publisher’s Note

All claims expressed in this article are solely those of the authors and do not necessarily represent those of their affiliated organizations, or those of the publisher, the editors and the reviewers. Any product that may be evaluated in this article, or claim that may be made by its manufacturer, is not guaranteed or endorsed by the publisher.
