# Supplementary material for: Therapeutic Suggestions During General Anesthesia Reduce Postoperative Nausea and Vomiting in High-Risk Patients – A Post hoc Analysis of a Randomized Controlled Trial
Source: Front Psychol. 2022 Jul 15;13:898326. doi: 10.3389/fpsyg.2022.898326 (PMC9337244; doi:10.3389/fpsyg.2022.898326)
Supplement: Supplementary file 1 [file Data_Sheet_1.docx]

Supplementary Material

# Study protocol

The full study protocol can be accessed online: <http://anaesthesie.rub.de/files/files/protocols/intraop_sugg_study_protocol_1.6.pdf>

# Post-hoc power analysis

**Supplementary Table 1.** Post-hoc power analysis according the incidence of postoperative nausea and vomiting (PONV) after surgery.

|  | All patients | Patients at high risk for PONV^1^ |
| --- | --- | --- |
|  | n = 385 | n = 180 |
| **Early PONV (within first 2 h)** |  |  |
| Proportion in suggestion group | 0.183 | 0.172 |
| Sample size in suggestion group | 191 | 87 |
| Proportion in control group | 0.227 | 0.312 |
| Sample size in control group | 194 | 93 |
| ⍺ error | 0.05 | 0.05 |
| Power (1-ß error) | 0.242 | 0.654 |
| **Delayed PONV (2-24 h)** |  |  |
| Proportion in suggestion group | 0.241 | 0.207 |
| Sample size in suggestion group | 191 | 87 |
| Proportion in control group | 0.299 | 0.344 |
| Sample size in control group | 194 | 93 |
| ⍺ error | 0.05 | 0.05 |
| Power (1-ß error) | 0.316 | 0.605 |
| **PONV within 24 h** |  |  |
| Proportion in suggestion group | 0.309 | 0.322 |
| Sample size in suggestion group | 191 | 87 |
| Proportion in control group | 0.366 | 0.452 |
| Sample size in control group | 194 | 93 |
| ⍺ error | 0.05 | 0.05 |
| Power (1-ß error) | 0.285 | 0.500 |

Hours refer to timepoint after admission to recovery room. Post-hoc power analysis was calculated for two independent proportions based on a one-tailed Fisher’s exact test. ^1^ PONV: Postoperative nausea and vomiting.

# Text of intraoperative therapeutic suggestions

## Part A (19 min, followed by a 10 min break, repeated several times. Start after induction of anesthesia and ends with stop of volatile anesthetic)

You are sleeping sound and deep.

And you can relax and rest, recover and draw strength,

because you are safe now, well-protected.

Everything that you hear and see and feel contributes to your best care.

And that’s why you can completely concentrate on your body’s own way to heal itself.

And we are right by your side.

My voice will go with you, and you can focus on it,

because what I say to you is important for you,

is important for your well-being and your healing.

With every deep breath that the respirator takes care of,

so that you can save your strength for later,

You can take in fresh air, oxygen, and whatever else is good for you

and is of help to you.

And with every exhale you can get rid of all the used air

and can let go all that is disturbing or a burden to you.

Breathe out and let go.

And with every breath you take you can take in ease, confidence and strength for healing,

on and on.

The surgery is going well. Surgeon and anesthetist are very satisfied.

Everything is going according to plan,

very professional, organized, and smooth.

They sure know their trait. They know how to optimize your care.

Your surgeons are very experienced in this type of surgery.

They are highly focused and work with thorough care.

Everything is ready and set for an optimal treatment.

Those are the best conditions for a safe and successful surgery.

The anesthetist and the nurse are responsible for nothing else but your comfort and your safety.

They are not departing from your side,

until you have successfully and safely weathered this challenge.

They are trained and experienced to look after you and take good care of you.

You are well sheltered.

All medication and equipment are ready to serve you well,

to do the right and necessary things in all situations.

We can always provide something good for you.

All your essential bodily functions are continuously monitored and assessed.

This consisting beeping sounds of the monitor shows your smooth, rhythmic heartbeat.

Your blood pressure is strong and steady.

The most essential tasks you are performing yourself.

We healthcare guides just pay attention and care so that you and your body find optimal conditions.

As your mind is resting your body can concentrate fully on self-healing and self-protection.

All of your organs, your heart and your blood vessels, are working together

to ensure wellbeing, safety and healing.

(Indirect suggestion using another speaker):

Perfect! Your patient looks like he is really doing well.

So calm and stable. He is doing a good job.

So, this surely will result in quick and full healing.

And he will soon be on his feet again,

and can enjoy a successful surgery.

Are you satisfied?

Completely! This has been going perfectly.

Great!

Listen to what I am saying! You already know that this surgery is good for you,

and will continue to help you.

And while your treatment here proceeds steady and thoroughly well,

everything is getting prepared for your optimal further care in the recovery room

and at the nurse’s station.

While your treatment is progressing perfectly you can retreat to an inner safe and comfortable place,

And you can recover and regain strength.

Your body knows that when your mind is calm and relaxed and confident

your unconscious mind can best take care for you.

There, all of the involuntary functions of your body are stored,

Your digestion, your fluid balance, your temperature regulation, your hormone release,

your program for wound healing, your infection defense, the rhythm of your sleep,

and everything that keeps you resilient and healthy.

And from right there, all of these functions are regulated and coordinated.

Your inner self takes perfect care of you.

Let`s now talk about the time after, after the surgery has been completed successfully.

Should you feel some pressure beneath the bandage, know that that`s quite normal,

and it just informs you that the healing has already begun.

It`s actually your white blood cells that have hurried to this place to do everything they can for the wound to

heal. They are summoning other cells, are sending out messengers, and are regulating blood circulation to make

sure that more nutrients, more oxygen, and more energy arrive there.

The signals your body sends from there translate into: Please don`t touch too much,

please leave it be for a while and let healing take place!

And then they need not to be so loud any longer and can become more and more quiet,

because everything has already been done to support your healing.

And everything is well taken care of.

You will notice that any tension or anxiety would only enhance pain.

Therefore, it might be better for you to relax, especially the part of the body that has undergone surgery. Just let

go.

It is quite normal to notice the area where surgery took place, and to feel sensations in the area where your body

is working hard to repair and put everything in order again.

It`s alright that all these signals and messages now become even fainter and softer and quieter.

As soon as you have noticed them your attention can move to other things. Like dropping your car off in a repair

shop and then going shopping or going for a walk.

It is good to know that your body and your cells exactly know what to do, and how to do it best.

Your attention is not even needed. You can turn your attention to something even more pleasant and joyful.

While your body and your caregivers take good care of everything,

you can recall the image of a comfortable and safe place,

while looking forward and being curious to what you might experience there,

and from time to time, maybe hear and feel from afar,

that someone is working diligently on your recovery.

And what else will be happening after your operation was completed beautifully?

Step by step all of your body function will start again:

Your blood pressure starts in full swing and your digestion.

You are producing saliva and you can swallow, and you can drink.

Everything returns in the right direction, always top down,

from the mouth to the stomach, and in the intestines and on and on,

uniformly in one direction, straight ahead.

And comfort can expand more and more, all over.

And you may ask yourself what you will want to eat first.

You can then send your blood circulation downwards to your intestines

who have rested in the meantime.

And with all the supply of energy and oxygen they gather pace again.

You can swallow fluids again,

And notice the fluids flowing down the esophagus into the stomach

and the stomach transports them further into the intestines

and in the intestines moving on, further and further,

consistently and continually in one direction, on and on.

And what else will be happening after your surgery is completed?

Oh yes, there were fears and worries. They were present for you to be careful and cautious,

and to protect you.

Now they are needless and without value,

Because everything is being done already

for your wellbeing, for your safety, and for your healing.

Your recovery is already happening, all by itself, unstoppable.

Your body is really good at this! You already know how to do it.

And we are by your side, and accompany you. You are safe now.

And one more thing for you to know, when the surgery is completed:

Since we supported your respiration for a long time

you can now take over your own breathing again.

Then, it is time to sigh and you can breathe deeply and strongly,

can clear your airways and cough, cough away all the mucus.

You will notice your strength returning.

We are all confident, and you too can be confident,

that you will be just fine after the surgery,

that the medication to relief nausea and pain are very effective,

and that you will be comfortable.

You will feel carefree and unaffected hours and days after the surgery

You will feel calm, warm, relaxed and content.

Whatever you experience will not disturb your wellbeing,

rather lets you know that your body is working eagerly to repair,

to put in place, to bring back into balance, and to normalize all functions.

Your healing can continue, growing steadily. On and on. Perfect!

## Part B (Length 12 min., for reorientation and recovery phase, start with termination of anesthetics, stop before extubation)

The surgery went well and is finishing up.

The healing has already begun.

You have rested for a while and have regained strength.

Now it is time to come back into the here and now.

All fears that were present to protect you are no longer necessary,

because you are safe now, and in good care.

All pain that was supposed to warn you is no longer necessary,

because you are getting all the support you need.

Any thoughts that could upset your stomach and digestion is unnecessary,

because everything is running normally and fine.

You are fully safe.

Your stomach and your digestion are working again.

Everything is moving in the right direction.

You can swallow freely to clear your throat from saliva.

You can look forward to drinking and eating.

What are you hungry for? It will certainly be delicious/

You can enjoy it, it will strengthen you.

Your abdomen is soft and tender, and feels great.

Bowel and kidneys are functioning normal.

The wound will heal quickly. Your body already knows how to regulate that beautifully.

You now can let out a sigh of relief, and breathe on your own again.

And with every deep breath

you can breathe in ease and strength,

and with every exhale

you can let go of any bother or burden, just breathing it away.

Notice how your strength is returning.

Imagine a stairway, leading you up, step by step, higher, higher and higher.

And with every step it seems brighter and fresher and the fog begins to lift.

Now draw your attention from the inside to the outside again.

Do you see the light? Hear the sounds?

You are awakening, more and more awake, and moving again.

You can reorient yourself and make sense of everything.

Everything is ok.

You are in good company and are well taken care of.

Everything around you is right there for your safety.

Your recovery and healing can continue, on and on.

The people around you are right there for you and take good care for you.

Your stay in the hospital will be pleasant.

It is encouraging and good to know to be attentively cared for,

protected, and thoughtfully looked after. Perfect!
